# LSD Administered as a Single Dose Reduces Alcohol Consumption in C57BL/6J Mice

**DOI:** 10.3389/fphar.2018.00994

**Published:** 2018-08-31

**Authors:** Kenneth Alper, Bin Dong, Relish Shah, Henry Sershen, K. Yaragudri Vinod

**Affiliations:** ^1^Department of Psychiatry, New York University School of Medicine, New York, NY, United States; ^2^Department of Neurology, New York University School of Medicine, New York, NY, United States; ^3^Division of Analytical Psychopharmacology, Nathan Kline Institute for Psychiatric Research, Orangeburg, NY, United States; ^4^Emotional Brain Institute, Orangeburg, NY, United States; ^5^Department of Neurochemistry, Nathan Kline Institute for Psychiatric Research, Orangeburg, NY, United States; ^6^Department of Child and Adolescent Psychiatry, New York University School of Medicine, New York, NY, United States

**Keywords:** lysergic acid diethylamide, psychedelic, hallucinogen, serotonin receptor agonists, alcohol, mouse, substance-related and addictive disorders

## Abstract

There is a substantive clinical literature on classical hallucinogens, most commonly lysergic acid diethylamide (LSD) for the treatment of alcohol use disorder. However, there has been no published research on the effect of LSD on alcohol consumption in animals. This study evaluated the effect of LSD in mice using a two-bottle choice alcohol drinking paradigm. Adult male C57BL/6J mice were exposed to ethanol to develop preference and divided into three groups of equal ethanol consumption, and then treated with single intraperitoneal injection of saline or 25 or 50 μg/kg LSD and offered water and 20% ethanol. The respective LSD-treated groups were compared to the control group utilizing a multilevel model for repeated measures. In mice treated with 50 μg/kg LSD ethanol consumption was reduced relative to controls (*p* = 0.0035), as was ethanol preference (*p* = 0.0024), with a group mean reduction of ethanol consumption of 17.9% sustained over an interval of 46 days following LSD administration. No significant effects on ethanol consumption or preference were observed in mice treated with 25 μg/kg LSD. Neither total fluid intake nor locomotor activity in the LSD-treated groups differed significantly from controls. These results suggest that classical hallucinogens in the animal model merit further study as a potential approach to the identification of targets for drug discovery and investigation of the neurobiology of addiction.

## Introduction

Alcohol-related disorders accounted for approximately 88,000 deaths annually and one in ten deaths in working age adults in the US from 2006 to 2010 (Stahre et al., 2014), and are estimated to cause 5.9% of deaths worldwide (World Health Organization, 2014). The 2012-2013 National Epidemiologic Survey on Alcohol and Related Conditions III found a 12-month prevalence of alcohol use disorder (AUD) of 13.9% (Grant et al., 2015), and the economic cost of alcohol misuse in the US in 2010 was estimated at $249 billion (Sacks et al., 2015).

A class of serotonin type 2A receptor (5-HT_2A_R) agonists with a distinctive domain of subjective effects, referenced as “classical hallucinogens” (Nichols, 2004), “serotonergic hallucinogens” (Halberstadt, 2015), or “psychedelics” (Nichols, 2016), are reported to be effective in some clinical studies of AUD (Krebs and Johansen, 2012; Bogenschutz et al., 2015), and other substance use disorders (Bogenschutz and Johnson, 2016). Among the classical hallucinogens, LSD, the prototype of this group of compounds, has the most extensive history of use in clinical research on AUD (Krebs and Johansen, 2012; Liechti et al., 2017). The literature on LSD for the treatment of AUD extends back to the 1950s, and a meta-analysis that included 6 randomized controlled trials (RCTs) involving a total of 536 subjects found that treatment with a single dose of LSD significantly reduced alcohol misuse for up to three months (Krebs and Johansen, 2012). This meta-analysis retrieved an additional 23 non-RCT open-label studies, case reports, or studies that did not otherwise meet inclusion criteria, indicating the considerable extent of the historical interest in clinical research on LSD for the treatment of AUD.

There is apparently no previously published work on the effects of LSD on the self-administration of alcohol or other abused substances in animals. In view of the elements of possible relevance to addiction in the signaling cascade downstream from the binding sites of LSD (Lopez-Gimenez and Gonzalez-Maeso, 2018), an effect of LSD on the self-administration of alcohol in animals would provide support for the animal model as a possible approach to target identification and drug discovery. This present research focuses on the effect of LSD on alcohol intake in a two-bottle choice drinking paradigm in C57BL/6J mice, an inbred alcohol-preferring strain (Melo et al., 1996).

## Materials and Methods

### Animals

This research was conducted in accordance with the National Institutes of Health and Nathan Kline Institute Animal Care and Use Committee’s guidelines. All experiments were conducted in adult male C57BL/6J mice (10–18 weeks old; Jackson Laboratory, United States). Mice were given unlimited access to standard mouse chow and water throughout the entire study.

### Ethanol Drinking Behavior

The effect of LSD on ethanol consumption was assessed using a two-bottle choice drinking paradigm. Mice were housed singly and habituated to reverse light-dark cycle for at least a week. The mice were then exposed to ethanol to develop preference with two bottles containing water and 20% ethanol offered for 24 h in the beginning of the dark phase for five days a week (Monday through Friday) for 4 weeks. The mice were then divided into three groups of equal ethanol intake based on the amount of ethanol consumed on the day before administration of either saline or LSD (25 or 50 μg/kg body weight, i.p.) once before the onset of the dark cycle. LSD was supplied by the NIDA Drug Supply Program, Bethesda, MD, United States. Water bottles were replaced with bottles containing water and ethanol after 10 min of treatment. Amounts of ethanol and water consumption were measured every 24 h. The positions of water and ethanol bottles were alternated every day to avoid place preference. Two bottles containing water and 20% ethanol were placed in a cage without a mouse to control for spillage and evaporation.

### Locomotor Activity

Ambulatory activity was measured as ambulatory counts (interruption of the total number of beams, on both the x and y-axis) with an infrared beam-based activity sensor (ATM3; Columbus Instrument, Columbus, OH, United States) over a 24-h period beginning at the dark cycle at 1 and 8 days after the administration of 50 μg/kg LSD or saline to ethanol-naïve mice.

### Statistical Analysis

Statistical analyses were performed with SAS^®^ software Version 9.4, utilizing a multilevel model (MLM) for repeated measures with PROC MIXED (SAS Institute Inc., 2018), and *t*-tests (one-tailed) with *p*-value = 0.05 as the threshold for significance.

## Results

The interval of observation of ethanol intake was continued until self-administration amounts of ethanol had reached equivalence, defined as two consecutive days on which ethanol consumption in the LSD-treated group equaled or exceeded the control group. For the group treated with 25 μg/kg, equivalence was reached at day 23; for the group treated with 50 μg/kg LSD it had not been reached at the time the study was terminated on day 46. For the group treated with 25 μg/kg LSD, the MLM independent variables included time, group and the interaction between them (to account for the change over time in the treated group). For the group treated with 50 μg/kg LSD, the interaction was not included because there was little evidence of change over time.

**FIGURE 1:**
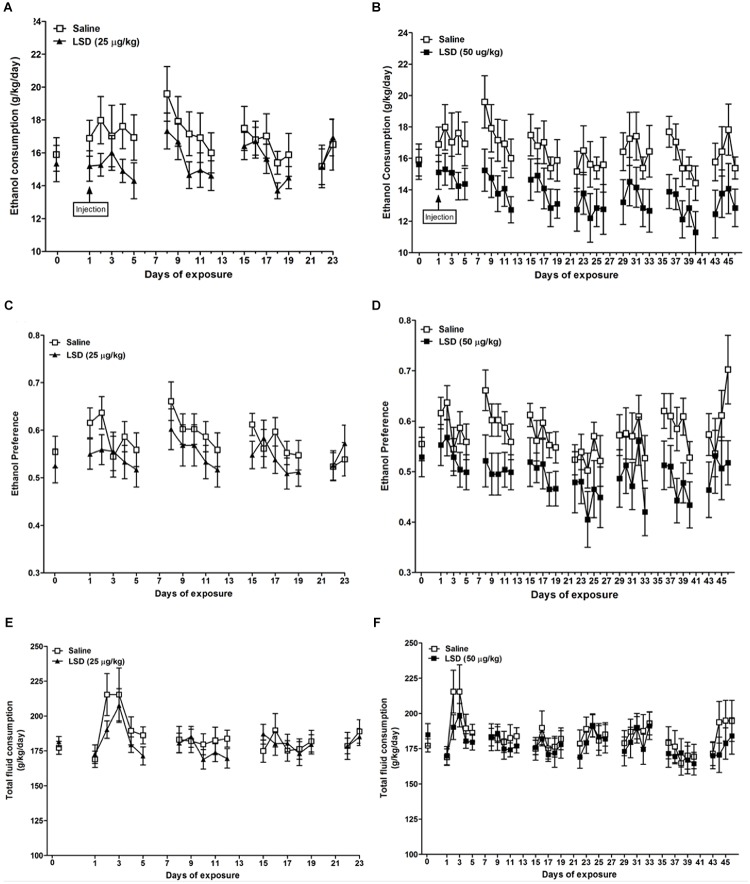
Ethanol consumption **(A,B)**, ethanol preference **(C,D)**, and total fluid consumption **(E,F)** in mice treated with single dosages of LSD of 25 (left column) or 50 (right column) μg/kg body weight, i.p. Data are presented as mean ± SEM (*n* = 8–10 per group). Horizontal axis indicates the number of days following the administration of LSD. The mice were divided into three groups of equal ethanol consumption based on the amount of ethanol consumed on the day before LSD administration (day 0). LSD dosages were administered on day 1 [“injection,” as indicated in the top row panels **(A,B)**]. In the group treated with 50 μg/kg LSD, MLM for repeated measures indicated significant reductions versus saline-treated controls in ethanol consumption (*p* = 0.0035) and preference (*p* = 0.0024). Differences from control were not significant for the group treated with 25 μg/kg. Total fluid intake did not differ significantly from controls in either LSD-treated group **(E,F)** (See “Results” in text).

Ethanol consumption was decreased relative to controls in the group treated with 50 μg/kg LSD (*F* = 11.99, df = 15, *p* = 0.0035; **Figure 1B**), without a significant effect with 25 μg/kg LSD (*F* = 3.08, df = 17, *p* = 0.0974; **Figure 1A**). Notably, the effect appears to have been sustained across the 46-day interval of observation. Likewise, ethanol preference, defined as ethanol consumption/total fluid intake, (where total fluid is defined as 20% ethanol solution + water) was decreased relative to controls in the 50 μg/kg LSD group (*F* = 13.32, df = 15, *p* = 0.0024; **Figure 1D**), with no difference from control for the group treated with 25 μg/kg LSD (*F* = 0.53, df = 17, *p* = 0.475; **Figure 1C**). Total fluid intake did not differ from control in either the 25 μg/kg LSD (*F* = 0.68, df = 17, *p* = 0.4204; **Figure 1E**) or 50 μg/kg LSD (*F* = 1.30, df = 15, *p* = 0.2722; **Figure 1F**).

In the group treated with 50 μg/kg LSD, mean daily ethanol consumption was 13.62 ± 3.02 g/kg/day compared to 16.60 ± 3.52 g/kg/day (mean ± SD) in controls (*t* = -1.86, df = 15, *p* = 0.0414), and ethanol preference was 0.493 ± 0.104 versus 0.580 ± 0.091 in controls (*t* = -1.83, df = 15, *p* = 0.0436), a 17.9% reduction in the LSD-treated animals, without a significant difference in mean daily total fluid intake. The group treated with 25 μg/kg LSD did not differ from control group with regard to mean daily ethanol consumption or preference, or total fluid intake.

**FIGURE 2:**
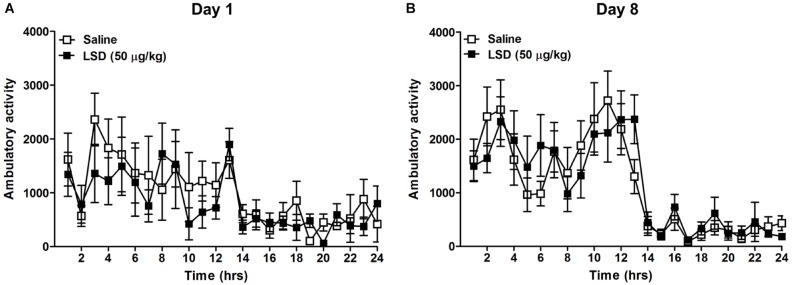
Home cage ambulatory activity (beams broken X-Y) was measured for 24 h following the administration of LSD 50 μg/kg weight, i.p. to ethanol-naïve mice on day 1 immediately following LSD administration **(A)** and 8 days later **(B)**. Data are presented as mean ± SEM (*n* = 8 per group). LSD administration did not significantly alter the ambulatory activity compared to saline treated mice on either day (See “Results” in text).

To evaluate a potential effect of LSD on ambulatory activity, a group of ethanol-naïve mice were treated with 50 μg/kg LSD or saline, and ambulatory counts were measured on day 1 and 8 (**Figure 2**). The result showed no significant difference in locomotor activity comparisons between control versus the LSD-treated group utilizing a MLM for hourly ambulatory counts on day 1 (*F* = 0.74, df = 14, *p* = 0.4054) or day 8 (*F* < 0.01, df = 14, *p* = 0.9907).

## Discussion

This study found reductions in both ethanol consumption and preference in the group of mice treated with the LSD dosage of 50 μg/kg. Although the magnitude of effect on ethanol consumption was limited, with a group mean reduction of 17.9%, it was sustained across a time interval of 46 days. Total fluid intake and locomotor activity did not differ among the control and LSD-treated groups, indicating that the observed effect on ethanol self-administration was not an artifact of a global effect on water or total fluid intake or possible differences in physically approaching or self-administering fluid.

This is apparently the first study to report on the effects of LSD on the self-administration of alcohol in animals. The literature reporting on the effect of classical hallucinogens on animal drug or alcohol self-administration is apparently limited to a single study that found the classical hallucinogen 1-(2,5-dimethoxy-4 iodophenyl)-2-aminopropane (DOI) administered intraperitoneally 15 min prior to a 12 h test session diminished alcohol preference in a two-lever water versus ethanol choice procedure in rats (Maurel et al., 1999). However, DOI is not frequently used by humans and has some features such as a very long half-life that would limit its generalizability to LSD, and the available evidence raises questions regarding its safety in humans (Kowalski and Salzman, 2012) relative to that of LSD (Nichols and Grob, 2018).

Mechanisms that could logically be proposed to explain the findings reported here include effects on G-protein coupled signal transduction and downstream effectors, second messengers, and gene expression (Halberstadt, 2015; Nichols, 2016; Lopez-Gimenez and Gonzalez-Maeso, 2018; Martin and Nichols, 2018), and a systematic exploration of these possibilities is well beyond the scope of this discussion. Classical hallucinogens produce 5-HT_2A_R desensitization (Nichols, 2016), and it has been suggested that persistently reduced signaling in downstream pathways linked to the 5-HT_2A_R might explain sustained effects observed in clinical studies on substance use (Bogenschutz and Johnson, 2016) or mood and anxiety symptoms (Vollenweider and Kometer, 2010; Ross et al., 2016).

Diminished signaling in downstream pathways linked to the 5-HT_2A_R could increase transmission via metabotropic glutamate receptors 2 and 3 (mGluR2/3) due to the reciprocally inhibitory relationship between 5-HT_2A_R and GluR2/3 (Nichols, 2016). Increased transmission through mGluR2/3, which are presynaptic and autoinhibitory on glutaminergic neurons, might be expected to diminish alcohol intake in view of evidence for mGluR2/3 agonists as possible pharmacotherapy for alcohol dependence (Goodwani et al., 2017; Windisch and Czachowski, 2018). Extracellular signal-regulated kinases 1 and 2 (ERK1/2) are positively coupled to the 5-HT_2A_R, suggesting that a diminution of 5-HT_2A_R signaling could have anti-addictive effects in view of evidence linking ERK1/2 activation to neuroadaptations and animal behaviors putatively associated with addiction (Girault et al., 2007; Pascoli et al., 2014).

Work that could logically follow includes neurochemical analysis aimed at identifying signaling pathways which might mediate the effect of LSD on alcohol intake observed in this study. Coupling among G proteins, phospholipase effectors and the 5-HT_2A_R subsumes a set questions of current interest in research on psychedelics. Although 5-HT_2A_Rs classically couple to Gα_q/11_, the effects of classical hallucinogens also apparently involve Gα_i/o_ (Lopez-Gimenez and Gonzalez-Maeso, 2018). G_i/o_ signaling interacts with brain region, for example alcohol intake in C57BL/6 mice is decreased by activation of G_i/o_ protein that is regionally expressed in the dorsal striatum (Robins et al., 2018). Investigative approaches to identifying the signaling pathways and cell populations involved may include measurement of protein expression and function as well as intracerebral injection of LSD in specific brain sites. Future studies may utilize alcohol-related behaviors such as drinking-in-the dark, intermittent access, or withdrawal to model AUD phenotypes (Leeman et al., 2010).

The finding of reduced ethanol preference with LSD in a rodent model suggests that the efficacy of LSD for the treatment of AUD reported in clinical studies may be not fully explained by psychological factors and involves a biologically mediated effect. The settings in which LSD has been administered as treatment for AUD are varied, including an RCT reporting positive results in which patients were strapped to a bed in a hospital room (Smart et al., 1966), suggesting that the effect of LSD on alcohol misuse may have involved a pharmacological determinant distinct from psychotherapeutic set and setting.

In view of the lack of prior published animal self-administration studies using LSD, reference to other animal behavioral paradigms may help contextualize the dosages of LSD used in this present research. Two behavioral paradigms appear relevant, the head twitch response (HTR) (Halberstadt and Geyer, 2013) and discrimination (Nichols, 2016). Reported ED_50_ dosages for correct responding to LSD in discrimination studies in mice range from approximately 170 to 200 μg/kg (Benneyworth et al., 2005; Winter et al., 2005; Krall et al., 2008). Similarly, in studies of the HTR, a rapid side-to-side rotational head movement in response to administered classical hallucinogens that is thought to correspond with compounds that produce psychedelic effects in humans, the maximum HTR to LSD is seen at 200 μg/kg, and 50 μg/kg appears to be approximately ED_50_ (Halberstadt and Geyer, 2013). The data reported from discrimination and HTR studies of LSD suggest that the dosages used in this study might not represent the maximum of the dose response curve, which should be addressed by evaluating higher dosages in future work.

In summary, this study found that LSD administered as a single dose reduces ethanol consumption and preference in C57BL/6J mice. These findings indicate that the apparent effect of LSD in AUD observed in clinical samples may be modeled in animals, suggesting the involvement of a biologically mediated determinant, and support the potential utility of classical hallucinogens in the animal model as a paradigm for target identification in drug discovery and investigation of the neurobiology of addiction.

## Data Availability Statement

The raw data supporting the conclusions of this manuscript will be made available by the authors, without undue reservation, to any qualified researcher.

## Author Contributions

KA originated the study and wrote the first draft of the manuscript. BD and RS performed the experiments. KA, HS, and KYV conceived and designed the study. KYV performed the experiments, wrote sections of the manuscript, and performed statistical analysis. All authors contributed to manuscript revision, read and approved the submitted version.

## Conflict of Interest Statement

The authors declare that the research was conducted in the absence of any commercial or financial relationships that could be construed as a potential conflict of interest.
